# Pharmacokinetic modeling of a novel hypoxia PET tracer [^18^F]HX4 in patients with non-small cell lung cancer

**DOI:** 10.1186/s40658-016-0167-y

**Published:** 2016-12-12

**Authors:** E. E. Verwer, C. M. L. Zegers, W. van Elmpt, R. Wierts, A. D. Windhorst, F. M. Mottaghy, P. Lambin, R. Boellaard

**Affiliations:** 1 Department of Radiology & Nuclear Medicine, VU University Medical Center, PO Box 7057, 1007 MB Amsterdam, The Netherlands; 2 Department of Nuclear Medicine & Molecular Imaging, Massachusetts General Hospital, Harvard Medical School, Boston, USA; 3 Department of Radiation Oncology (MAASTRO), GROW–School for Oncology and Developmental Biology, Maastricht University Medical Center, Maastricht, The Netherlands; 4 Department of Nuclear Medicine, Maastricht University Medical Center, Maastricht, The Netherlands; 5 Department of Nuclear Medicine, University Hospital RWTH Aachen, Aachen, Germany; 6 Department of Nuclear Medicine and Molecular Imaging, University Medical Center Groningen, Groningen, The Netherlands

**Keywords:** ^18^F-3-Fluoro-2-(4-((2-Nitro-1H-Imidazol-1-yl)Methyl)-1H-1,2,3-Triazol-1-yl)Propan-1-ol ([^18^F]HX4), Hypoxia, Molecular imaging, Positron emission tomography (PET), Non-small cell lung cancer (NSCLC), Tracer kinetic modeling, Standardized uptake value (SUV), Tumor-to-blood ratio, Image-derived input function (IDIF)

## Abstract

**Background:**

[^18^F]HX4 is a promising new PET tracer developed to identify hypoxic areas in tumor tissue. This study analyzes [^18^F]HX4 kinetics and assesses the performance of simplified methods for quantification of [^18^F]HX4 uptake.

To this end, eight patients with non-small cell lung cancer received dynamic PET scans at three different time points (0, 120, and 240 min) after injection of 426 ± 72 MBq [^18^F]HX4, each lasting 30 min. Several compartment models were fitted to time activity curves (TAC) derived from various areas within tumor tissue using image-derived input functions.

**Results:**

Best fits were obtained using the reversible two-tissue compartment model with blood volume parameter (2T4k+V_B_). Simplified measures correlated well with V_T_ estimates (tumor-to-blood ratio (TBr) *R* ^2^ = 0.96, tumor-to-muscle ratio *R* ^2^ = 0.94, standardized uptake value *R* ^2^ = 0.89).

**Conclusions:**

[^18^F]HX4 shows reversible kinetics in tumor tissue: 2T4k+V_B_. TBr based on static imaging at 2 or 4 h can be used for quantification of [^18^F]HX4 uptake.

**Electronic supplementary material:**

The online version of this article (doi:10.1186/s40658-016-0167-y) contains supplementary material, which is available to authorized users.

## Background

Tumor tissue often exhibits insufficient or abnormal vasculature, leading to reduced delivery of oxygen and nutrients to tumor cells. Decreased pO_2_ tension (“hypoxia”) can lead to upregulation of hypoxia inducible factor-1 (HIF-1), promoting tumor cell survival as well as tumor growth and metastatic potential [1]. Tumor targeted therapy strategies, such as radiation therapy and many types of chemotherapy, require the presence of oxygen in the cell [2, 3]. Tumor hypoxia is therefore an important predictor of overall survival and response to therapy [4]. Advanced treatment strategies for specifically targeting hypoxic tumor cells [5, 6], such as hypoxia-specific chemotherapy [7], radiosensitizers [8], or radiation dose painting [9–11], are currently being evaluated in clinical trials. For correct diagnosis as well as successful application of these advanced treatment strategies, accurate assessment of oxygenation status and the heterogeneous distribution of hypoxia in tumor tissue are required.

The gold standard for assessing tissue oxygenation is the polarographic needle electrode. However, this method is invasive and limited to accessible lesions. Furthermore, it is not suitable for visualizing the heterogeneous distribution of hypoxia within the tumor, which would be required for radiation therapy dose painting. Positron emission tomography (PET) allows for non-invasive in vivo imaging of tumor tissue characteristics, making this technique suitable for both identification of the presence of hypoxia as well as for assessing the heterogeneous distribution.

Several hypoxia-specific PET tracers have been developed. Most are based on a nitroimidazole group which is bioreduced upon entering the cell and retained in hypoxic cells, when rapid re-oxidation does not occur. The most evaluated hypoxia tracer is ^18^F-fluoromisonidazole ([^18^F]FMISO) [12], which shows slow clearance from normoxic tissue, likely due to its lipophilicity, necessitating long time intervals between injection of the tracer and imaging.

The novel nitroimidazole-based PET tracer, 3-[^18^F]fluoro-2-(4-((2-nitro-1H-imidazol-1-yl)methyl)-1H-1,2,3-triazol-1-yl)propan-1-ol ([^18^F]HX4), is reported to be relatively hydrophilic [13]. This characteristic facilitates fast clearance from normoxic tissues, thus enhances image contrast [14]. [^18^F]HX4 also shows favorable characteristics with respect to robustness; metabolite formation is reported to be very limited, with parent fractions of 82% remaining after 2 h in human plasma [15].

The hypoxia specificity of [^18^F]HX4 uptake has been verified in a preclinical setting [13]. However, to be able to use PET imaging to assess tumor hypoxia levels, quantification of tracer uptake in the tissue is required. Full pharmacokinetic modeling using dynamic PET imaging can be used to accurately quantify the tracer uptake. However, due to the elaborate imaging protocol required for full pharmacokinetic modeling, a simplified method (such as the standardized uptake value (SUV) which is based on a PET image acquired with a static imaging protocol) is often preferred in a routine clinical setting. In fact, several published papers on [^18^F]HX4 use these simplified methods for quantification of tracer uptake [16–19]. The validity of these simplified methods has, however, not yet been established for [^18^F]HX4. The aim of the current paper was to study the kinetics of [^18^F]HX4 using pharmacokinetic modeling and to assess the validity of several commonly used simplified parameters for quantifying [^18^F]HX4 uptake by comparing their performance to full pharmacokinetic modeling results. To this end, dynamic PET studies, each study consisting of three 30-min dynamic PET scans and spanning of 4.5 h in total, were obtained from patients diagnosed with non-small cell lung cancer (NSCLC) and analyzed.

## Methods

### Inclusion criteria

Patients were recruited from the PET-Boost trial (NCT01024829) [20]. Included were patients with pathologically proven NSCLC stages T2-4, N0-3, and M0. Further inclusion criteria were minimal primary tumor diameter of 4 cm, ECOG performance status ≤2, adequate organ function, and FDG-PET standardized uptake value (SUV) ≥5 for the primary tumor. Exclusion criteria were radiotherapy to the thorax prior to PET/CT imaging, tumor growth in large blood vessels, and post-obstructive atelectasis or infiltration that cannot be distinguished from tumor tissue on a PET/CT scan. The study was approved by the competent Medical Ethical Review Committee, and each patient signed informed consent after verbal and written explanation.

### Synthesis of [^18^F]HX4

The precursor for the synthesis of [^18^F]HX4 was provided by the Threshold Pharmaceuticals, San Francisco, USA. Cyclotron-produced ^18^F was worked up under standard conditions with Kryptofix [2.2.2] and K_2_CO_3_ and used for the radiolabeling of [^18^F]HX4 at 110 °C for 5 min in 1 mL of a 50/50 mixture of *t-*butanol/acetonitrile. Subsequent acidic hydrolysis with HCl (5 min at 105 °C) yielded the product. [^18^F]HX4 was purified with semiprep HPLC (ACE 5 AQ, 250 × 10 mm, 10 μm, eluent: water/acetonitrile/formic acid 95/5/0.1). The fraction containing the product was isolated and diluted with 20 mL of water for injection and passed over a Sep-Pak HLB cartridge (Waters, Etten-Leur, The Netherlands) to trap the product. After washing the cartridge with 10 mL of water for injection, the product was eluted with 1 mL of sterile ethanol and 10 mL of a solution of 7.1 mM NaH_2_PO_4_ in saline (pH 5.2) through a Millex GV 0.22 μm filter (Merck Millipore, Darmstadt, Germany) into a sterile vial to yield a sterile and pyrogen-free solution with radiochemical purity >97% and specific activity 11–136 GBq/μmol at time of injection.

### Data acquisition

PET/CT imaging was performed on a Gemini TF64 PET/CT scanner (Philips Healthcare, Cleveland, USA) with spatial resolution ~7 mm FWHM. For each scan, the patient was positioned on a flat tabletop using a laser alignment system with both arms above the head in a dedicated radiotherapy arm-support system. First, a 30-min dynamic PET scan was acquired, starting simultaneously with intravenous injection of 426 ± 72 MBq [^18^F]HX4 [16]. Afterwards, the patient returned for two additional 30 min dynamic scans at 2 and 4 h p.i. Each PET scan was preceded by a low dose CT with matrix dimensions of 512 × 512 × 45 voxels (1.17 × 1.17 × 4.00 mm^3^) for attenuation and scatter correction purposes and anatomical information. PET data were corrected for decay, dead time, attenuation, and scatter and reconstructed using a three-dimensional row action maximum likelihood reconstruction algorithm (3-D RAMLA) into 32, 6, and 6 frames, respectively (10 × 6 s, 6 × 20 s, 4 × 30 s, 5 × 60 s, 5 × 120 s, 2 × 300 s; 6 × 300 s; 6 × 300 s) of matrix dimensions 144 × 144 × 45 voxels (4 × 4 × 4 mm^3^). All data were reconstructed in concordance with guidelines for quantitative [^18^F] fluorodeoxyglucose PET studies [21, 22].

PET images at 2 and 4 h p.i. were co-registered to the last PET frame of the first dynamic scan (acquired 25–30 min p.i.) using a rigid co-registration algorithm in the software program Vinci (version 2.56.0; Max Planck Institute for Metabolism Research, Cologne, Germany). The three co-registered PET scans were then concatenated to provide one dynamic PET scan with a total time span of 4.5 h. Accuracy of co-registration results and absence of frame-by-frame motion was verified by visually inspecting the full scan and time activity curves (TAC).

### Kinetic analysis

#### Extraction of time activity curves

For each patient, up to five volumes of interest (VOI) were defined manually within the largest primary tumor structure. One whole-tumor VOI was defined on the low dose CT of the first PET/CT scan (VOI^CT^). Additionally, VOI were defined on the averaged PET image acquired over time interval 4–4.5 h p.i.; to investigate possible differences in kinetics within tumor tissue, up to three VOI were defined in areas showing relatively high (VOI^High^), average (VOI^Avg^), and low (VOI^Low^) PET signal, as determined visually with the aid of thresholding. In addition, as most tumors featured an area with markedly low PET signal, possibly indicating necrosis, a whole-tumor VOI was defined where this area was excluded (VOI^Viable^). For comparison, VOI in several healthy tissues (muscle, lung, fat, and liver tissue; when visible within the PET field of view) were defined manually onto the low dose CT image, taking care to avoid tissue boundary limits. All VOI were defined by the same person and manually adjusted to exclude large blood pool structures identified on early PET frames showing the tracer in the arterial blood vessels. VOI were then projected onto the full dynamic PET scan to derive TAC.

#### Derivation of plasma input functions

For each patient, whole-blood TAC were derived from a VOI that was defined manually within the ascending aorta, as identified using an early PET frame best displaying the blood pool. All VOI were defined by the same person, and care was taken to obtain large VOI (2.28 ± 0.99 cm^3^) whilst avoiding vessel boundaries. To derive metabolite-corrected image-derived input functions (IDIF), the whole-blood TAC were interpolated and corrected using population-based plasma-to-blood ratios and parent fraction data obtained from an internal report, provided by Doss et al. [15], containing data from three healthy volunteers for several time points up to 2 h p.i. As plasma-to-blood ratios were reported to be equal to 1 and stable over time, correction for plasma-to-blood ratio was omitted. Metabolite formation over time was estimated by fitting a Hill function through all parent fraction data points reported. Whole-blood TAC were then corrected for metabolites by multiplication with the resulting parent fraction curve. As parent fraction data was limited to the first 2 h p.i., the accuracy of parent fraction estimates extrapolated to 4 h p.i. might be reduced. Therefore, the analysis was conducted twice, once for TAC spanning time interval 0–2.5 h p.i. and once for time interval 0–4.5 h p.i. In addition, to assess the impact of metabolite correction, the analyses were repeated with non-corrected IDIF (IDIF_WB_).

#### Non-linear regression analysis

Several standard compartment models were fitted to all tissue TAC using non-linear regression routines (NLR) using kinetic analysis software developed in MatLab (MathWorks) at VU University Medical Center [23]. Models evaluated were the reversible single-tissue compartment model (1T2k) and the irreversible and reversible two-tissue compartment model (2T3k and 2T4k, respectively). Each model was evaluated with and without correction for blood volume fraction (V_B_). Boundary conditions for all estimated kinetic parameters were applied to prevent errors due to local cost function minima. In multiple runs, parameter ranges were decreased from [0.100] to the following intervals: K_1_ [0.1], k_2_ [0.1], k_3_ [0.0.5], k_3_/k_4_ [0.5], and V_B_ [0.0.25], yielding the results reported in this paper. To account for noise level, residual sum of squares were weighted to $\frac{L^2}{T}{e}^{-2\lambda t}$, where *L* is the frame duration, *T* is the estimated total number of trues, *λ* is the decay constant, and *t* is the frames mid time).

To investigate the effect of study duration, we have fully analyzed the dataset twice, once including only data acquired over the first 0–2.5 h p.i. and once including the full 0–4.5 h data. In this paper, we will show the results for the 0–2.5 h dataset and discuss the effect of including the 4–4.5 h data in the analysis.

The model best suited to describe the kinetics of [^18^F]HX4 in tumor tissue was selected based on the Akaike information criterion (AIC) for small sample sizes [24, 25] for all tumor VOI.

#### Validation of simplified parameters

Simplified parameters, e.g., standardized uptake value (SUV), using various normalization factors (body weight (BW), lean body mass (LBM), body surface area (BSA) and body mass index (BMI)), tumor-to-blood ratio (TBr), and tumor-to-muscle ratio (TMr), were calculated from the averaged images of both the 2 h p.i. as well as the 4 h p.i. PET scan. For all tumor VOI, these measures were then compared to quantitative pharmacokinetic parameters derived with full kinetic modeling.

## Results

PET/CT scans from 8 patients (5 male, 3 female) were analyzed. Pathology indicated adenocarcinoma (*N* = 4), squamous cell carcinoma (*N* = 3), and large cell carcinoma (*N* = 1). Mean age of the patients was 64 years (range 51–82 years), mean weight 75 kg (range 56–93 kg), mean height 172 cm (range 154–180 cm), and mean size of the CT-based whole-tumor VOI was 111 mL (range 29–208 mL). Injected activity was 426 ± 72 MBq. All patients had been previously treated with at least one course of chemotherapy, but PET/CT imaging was performed before the onset of external beam radiation therapy.

**Fig. 1:**
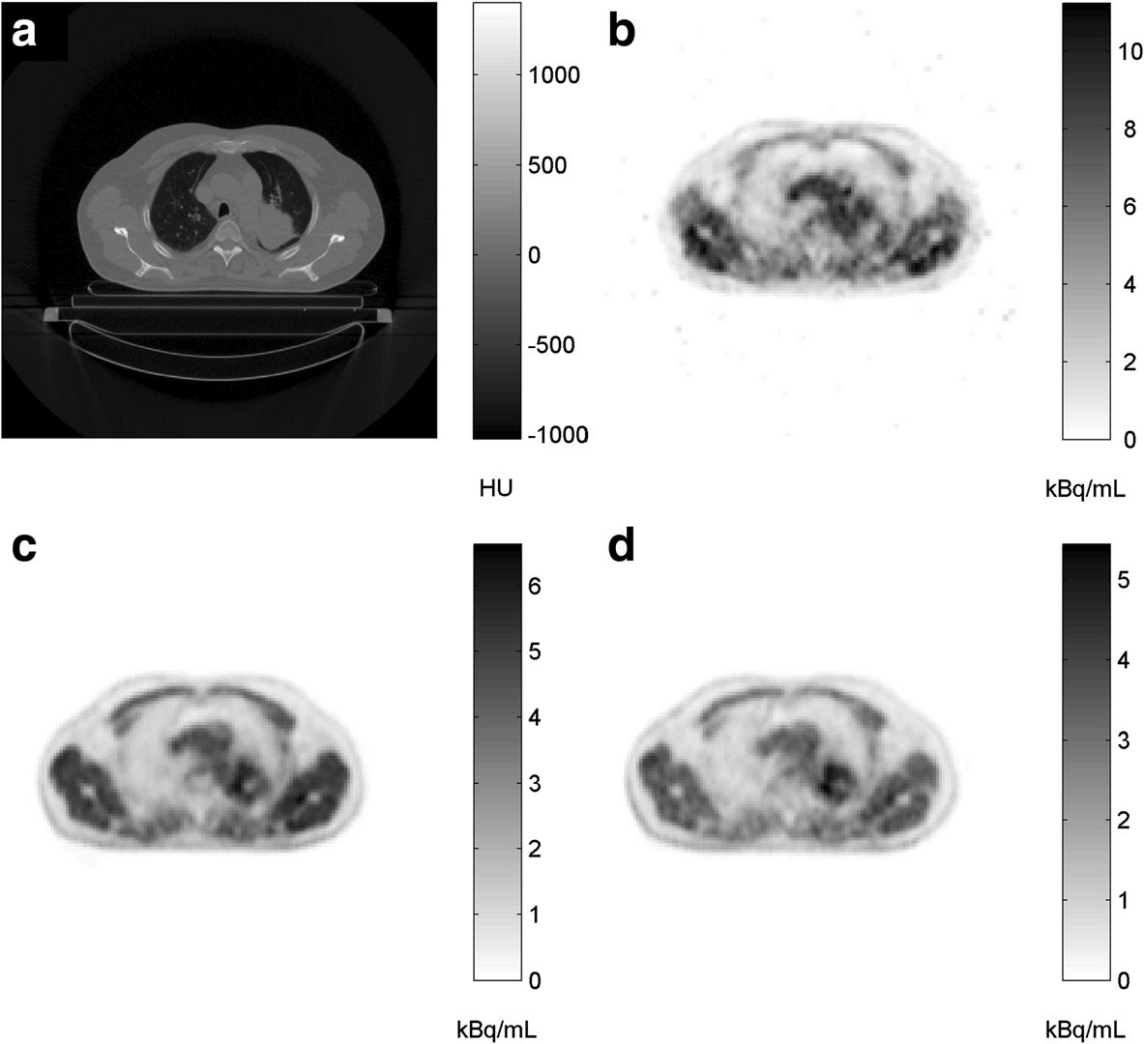
Typical [^18^F]HX4 PET/CT images of a patient with NSCLC: **a** low dose CT; **b**, **c**, **d** averaged PET image acquired over 25–30 min p.i., 119–149 min p.i., and 237–267 min p.i., respectively

Typical examples of acquired data are given in Fig. 1 (Additional File 1 shows the VOI in these images). Heterogeneous [^18^F]HX4 uptake in tumor tissue was observed. SUV_BW_ and tissue-to-muscle and tissue-to-blood ratios at 2 and 4 h p.i. are shown in Table 1 for various areas within the tumor and for several healthy tissues. High correlation was found between tumor activity concentrations at 4 and 2 h (*R* ^2^ = 0.93; ICC = 0.86).

**Table 1:** Study group averages of SUV_BW_ and tissue-to-muscle (TiMr) and tissue-to-blood (TiBr) ratios (±2 SD) at 2 h and 4 h p.i. for various tissues

Tissue type	SUV_BW_		TiMr		TiBr	
	2 h	4 h	2 h	4 h	2 h	4 h
Fat	0.27 ± 0.07	0.18 ± 0.08	0.29 ± 0.05	0.28 ± 0.09	0.28 ± 0.06	0.27 ± 0.10
Liver	1.28 ± 0.40	1.02 ± 0.23	1.54 ± 0.61	1.93 ± 1.12	1.48 ± 0.62	1.88 ± 0.89
Lung	0.24 ± 0.11	0.16 ± 0.10	0.25 ± 0.08	0.24 ± 0.12	0.24 ± 0.08	0.23 ± 0.09
Muscle	0.95 ± 0.28	0.67 ± 0.34	–	–	0.96 ± 0.18	0.98 ± 0.28
Tumor	0.93 ± 0.56	0.72 ± 0.56	0.98 ± 0.46	1.09 ± 0.82	0.93 ± 0.35	1.02 ± 0.45
Tumor, viable	1.07 ± 0.63	0.86 ± 0.67	1.12 ± 0.49	1.29 ± 0.98	1.07 ± 0.36	1.21 ± 0.52
Tumor, high	1.22 ± 0.75	1.04 ± 0.83	1.28 ± 0.71	1.61 ± 1.58	1.22 ± 0.54	1.50 ± 0.96
Tumor, mid	0.96 ± 0.66	0.74 ± 0.54	1.00 ± 0.55	1.12 ± 0.79	0.95 ± 0.43	1.05 ± 0.39
Tumor, low	0.39 ± 0.52	0.33 ± 0.40	0.43 ± 0.59	0.62 ± 1.04	0.41 ± 0.51	0.55 ± 0.70

### Pharmacokinetic modeling

**Fig. 2:**
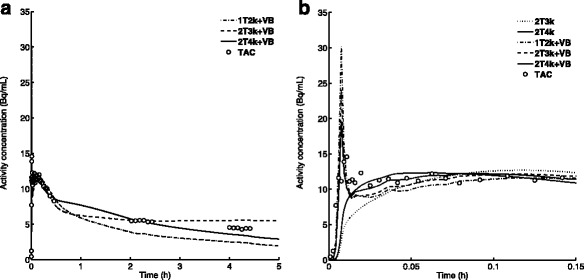
Non-linear regression fits to a tumor time activity curve from the typical example displayed in Fig. 1 using several of the models evaluated. **a** Fits to 2.5 h TAC extrapolated to 5 h p.i. **b** Detailed view of the first 9 min p.i.

AIC results are summarized in Table 2 for the 2.5 h dataset, analyzed with IDIF. Results indicate reversible kinetics in tumor tissue with governing model 2T4k+V_B_. Typical examples of NLR fits to the “viable” whole-tumor TAC (0–2.5 h p.i.) are shown in Fig. 2 for several models evaluated. Inclusion of 4–4.5 h data did not change the overall model preference (Additional File 2), and 2T4k+V_B_ fits derived were equivalent to 2T4k+V_B_ derived from 2.5 h data, with similar V_T_ values (*R* ^2^ = 0.96). At 4 h p.i., the 2T4k+V_B_ fit underestimated activity concentrations compared to measured TAC (VOI^Viable^) by 14.7 ± 7.7%, while the 2T3k+V_B_ fit overestimated by 10.2 ± 7.4%. Omission of the population-based metabolite correction did not lead to changes in model selection but led to a systematic 15% decrease in NLR-derived V_T_ values. NLR estimates of activity concentrations at late time points were only marginally affected; activity concentration estimates at 4 h p.i. obtained with metabolite correction were 0.74% higher for the 2T3k+V_B_ model and 0.09% lower for the 2T4k+V_B_ model than those obtained without metabolite correction.

**Table 2:** VOI size (cm^3^) and model preference (%) for [^18^F]HX4 kinetics in various tissues according to AIC, based on the 2.5 h dynamic PET data

		Model					
Tissue type	VOI size (cm^3^)	1T2k	2T3k	2T4k	1T2k+V_B_	2T3k+V_B_	2T4k+V_B_
Fat	3.7 ± 1.1	–	–	25	12.5	50	12.5
Lung	20.1 ± 9.3	–	–	–	37.5	50	12.5
Muscle	4.5 ± 2.5	–	12.5	25	62.5	–	–
Liver	17.1 ± 9.2	–	25	75	–	–	–
Tumor	111.4 ± 64.1	–	–	–	–	–	100
Tumor, viable	48.0 ± 27.2	–	–	–	–	–	100
Tumor, high	3.0 ± 2.8	–	–	25	–	12.5	62.5
Tumor, mid	1.8 ± 0.8	–	12.5	25	–	37.5	25
Tumor, low	1.1 ± 0.2	–	–	–	–	75	25

For clarity, values of 0% are not shown

### Performance of simplified methods

**Fig. 3:**
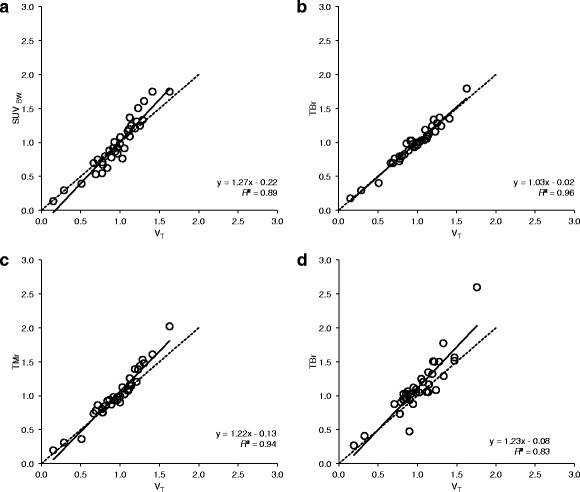
**a**, **b**, **c** Averaged simplified measures at 2–2.5 h p.i. for all tumor VOI, compared to V_T_ derived with NLR from 0–2.5 h dynamic PET data using the 2T4k+V_B_ model and IDIF: **a** standardized uptake value normalized to patient weight (SUV_BW_), **b** tumor-to-blood ratio (TBr), **c** tumor-to-muscle ratio (TMr); **d** TBr as measured at 4–4.5 h p.i. compared to V_T_ derived from 0–4.5 h dynamic PET data

For tumor tissue, simplified parameters correlated well to V_T_ derived with 2T4k+V_B_, as shown in Fig. 3. Best results for 2.5 h data were obtained with TBr (*R* ^2^ = 0.96), followed by TMr (*R* ^2^ = 0.94). SUV also performed well for all normalization factors investigated (SUV_BW_ *R* ^2^ = 0.89, SUV_BSA_ *R* ^2^ = 0.91, SUV_LBM_ *R* ^2^ = 0.82, SUV_BMI_ *R* ^2^ = 0.88). The 4.5 h dataset yielded lower correlations (TBr *R* ^2^ = 0.83, TMr *R* ^2^ = 0.74, SUV_BW_ *R* ^2^ = 0.76, SUV_BSA_ *R* ^2^ = 0.78, SUV_LBM_ *R* ^2^ = 0.67, SUV_BMI_ *R* ^2^ = 0.84).

## Discussion

In this study, [^18^F]HX4 kinetics over the course of 4.5 h after injection were studied using dynamic PET imaging and pharmacokinetic modeling. Results indicate that a reversible two-tissue compartment model best describes [^18^F]HX4 kinetics in tumor tissue. Good correlation was found between simplified parameters and V_T_, indicating that static imaging at 2 or 4 h p.i. is a suitable alternative to full pharmacokinetic modeling for quantifying [^18^F]HX4 uptake.

Theoretically, the uptake mechanism of nitroimidazole would indicate an irreversible model. Therefore, for [^18^F]FMISO, generally, an irreversible model is used for quantification [26]. The data-driven method used for model selection in this paper found a reversible model for [^18^F]HX4. This apparent discrepancy may be due to differences in partition coefficient. [^18^F]HX4 is more hydrophilic than [^18^F]FMISO, leading to faster diffusion and clearance rates. This hypothesis is corroborated by the fact that our results are consistent with pharmacokinetic modeling results published for the hypoxia tracer [^18^F]FAZA [27], which, like [^18^F]HX4, is relatively hydrophilic compared to [^18^F]FMISO. Nevertheless, the apparent discrepancy begs the question whether the tumor tissue analyzed was in fact hypoxic. Although no tissue pO_2_ levels were measured, as this procedure is highly invasive, the size of the tumors and presence of a core of limited to no uptake, does suggest the presence of hypoxic areas.

A possible explanation is that large VOI uptake kinetics could have been erroneously identified as reversible due to uptake heterogeneity causing an apparent reversible component to the averaged TAC [28]. For this reason, smaller VOI with different uptake levels were also evaluated, and also, these VOI kinetics were found to be reversible. It should be noted that for VOI in areas of relatively low uptake, model preference did seem to shift toward the 2T3k+V_B_ model. This is to be expected since, given the relatively small size of these VOI (Table 2) in combination with the low counts per voxel, the TAC will be noisier. This leads to smaller differences between the residual sums obtained with the two-tissue compartment models. In that case, AIC will indicate a preference for the model with the smaller number of parameters, i.e., the 2T3k+V_B_ model.

As shown in Fig. 2, for the typical example, fits obtained with 2T4k+V_B_ underestimate the 4.5 h p.i. activity concentrations in some patients. This may in part explain the lower correlations between simplified parameters and V_T_ that were observed for 4.5 h data. The discrepancy could be the result of small differences in image-derived activity concentration due to co-registration errors. Although a laser alignment system together with a dedicated arm-support system were used to ensure proper positioning, and co-registration results were visually verified, small co-registration discrepancies are likely to occur as the thorax area is highly flexible.

Another explanation could be the lack of data for the time intervals between scans. 4.5 h dynamic imaging was deemed too great a burden on the patient. Therefore, imaging was limited to three 30-min dynamic scans spanning 4.5 h. It is conceivable that the shape of the fits would have changed somewhat had the full 4.5 h TAC been available. However, assuming that the first 30 min data provides accurate estimates of K_1_ and k_2_, the 2T3k+V_B_ model could only fit the 4.5 h data by decreasing k_3_. This would lead to underestimation at 2 h p.i. The 2T4k+V_B_ model is better able to find a proper fit through the whole TAC.

In pharmacokinetic modeling, it is often assumed that metabolites remain in the blood, and only parent tracer is able to enter the tissue. Plasma input functions are therefore corrected for radiolabeled metabolites. However, for some tracers, metabolites may enter the tissue. As [^18^F]HX4 metabolite formation rate is slow, tissue metabolite accumulation in the tissue will be slow but could be large enough at 4 h to cause distinguishably higher tumor activity concentrations. As 0–2.5 h parent fraction data were extrapolated to 4.5 h p.i., it is possible that parent fraction estimates at 4.5 h p.i. were overestimated. This, however, would not explain the observed discrepancy between fit and TAC at 4.5 h p.i. As shown in the “Results” section, omitting metabolite correction entirely led to only marginal changes in 4 h activity concentration estimates. It is therefore unlikely that the inability of either model to fit the 4.5 h data is the result of underestimation of the metabolite fractions.

Theoretically, the discrepancy could be caused by a 3rd tissue compartment showing very slow irreversible trapping of the radiotracer. A (irreversible) three-tissue compartment would then yield closer fits to the data. However, given that the discrepancies between TAC and fit observed at 4 h p.i. are small, k_5_ would have to be so small that resulting NLR fits will be unreliable even if full 4.5 h dynamic data were available. Yet, based on the data presented in this study, possible influence of a third compartment cannot be excluded. Such a conclusion would require dynamic PET imaging over exceptionally long imaging times (>4.5 h), flawless co-registration of PET images, and voxel-by-voxel (to limit the influence of averaging and co-registration errors on shape of the TAC), for instance, using spectral analysis, a method that does not a priori assume a compartment model type [29]. To our best assessment, this type of study would be impossible to achieve in a clinical setting. Theoretically, the third compartment would represent the hypoxia-specific uptake mechanism. From the data, it is clear that contribution of this third compartment would be very small while the observed increase in TMr over time does indicate a marked difference in retention rates in certain tumor tissue areas compared to normoxic (muscle) tissue. This seems to suggest that the second compartment already contains a hypoxia-specific component (albeit reversible), and evaluation of hypoxia is possible with [^18^F]HX4 PET within the imaging time interval used of the study.

Since equivalent modeling results were found for the 2.5 h and 4.5 h dataset (*R* ^2^ = 0.96 for V_T_ values), and given the high correlation between simplified measures and V_T_ values, static imaging at 2 h p.i. may suffice for quantitative assessment of [^18^F]HX4 uptake. This would allow for a clinically more convenient imaging protocol. Yet, it should be noted that imaging at 4 h p.i. would yield higher image contrast, mainly because of better washout of [^18^F]HX4 in normoxic tissues, which may facilitate delineation of high [^18^F]HX4 uptake areas. The use of very late (4 h p.i.) uptake images could be of importance when [^18^F]HX4 PET imaging is performed for radiation oncology purpose (e.g., definition of biological target volumes), but is less critical for quantification of tumor V_T_. Performance of tumor delineation techniques is beyond the scope of this paper and will be part of future research.

IDIF were corrected for plasma-to-blood ratio and metabolites based on population averages based on data by Doss et al. [15]. Individual differences in metabolite formation and plasma-to-blood ratio are not taken into account. For tracers exhibiting high metabolite formation rate in combination with high variability between patients, individual parent fraction correction would be required. Metabolite formation for [^18^F]HX4 rate is shown to be slow in healthy subjects by Doss et al. [15]. Therefore, individual differences in metabolite formation rate will likely cause only marginal differences in parent fraction values. Furthermore, similar hypoxia PET tracers all exhibit similarly slow metabolite formation rates in humans: [^18^F]FMISO [30], [^18^F]FETNIM [31], [^18^F]EF5 [32], and [^18^F]FAZA [27]. Robustness of [^18^F]HX4 in patients should be confirmed in a larger study population. Nevertheless, as metabolites are assumed to remain in the blood stream, differences in metabolism would affect TBr and SUV values. It cannot be assumed that the effect is similar to its effect on V_T_ values derived with metabolite-corrected plasma input functions, which may in part explain the somewhat lower correlation between TBr and V_T_ for the 4.5 h dataset for which extrapolated metabolite data was used (Fig. 3d). However, the fact that correlations between TBr and SUV with V_T_ derived with uncorrected input functions were high, does suggest that differences in parent fractions and plasma-to-blood ratios between patients were small and would not affect the conclusions presented in this paper.

## Conclusions

[^18^F]HX4 kinetics can best be described by a reversible two-tissue compartment model. Most accurate simplified parameter is tumor-to-blood ratio. These quantitative metrics can be obtained without dynamic imaging, using a static (whole body) imaging protocol at either 2 or 4 h p.i.

## Additional files

Additional file 1: Visualization of VOI for the typical images displayed in Fig. 1. (PDF 3910 kb)
Additional file 2: Model preference (%) for [^18^F]HX4 kinetics in various tissues according to AIC, based on 4.5 h dynamic PET data. (PDF 59 kb)
