# Supplementary material for: Pharmacokinetic modeling of a novel hypoxia PET tracer [18F]HX4 in patients with non-small cell lung cancer
Source: EJNMMI Phys. 2016 Dec 12;3:30. doi: 10.1186/s40658-016-0167-y (PMC5153396; doi:10.1186/s40658-016-0167-y)

Additional Figure 1. Visualization of the various VOI defined onto the typical images displayed in Fig. 1

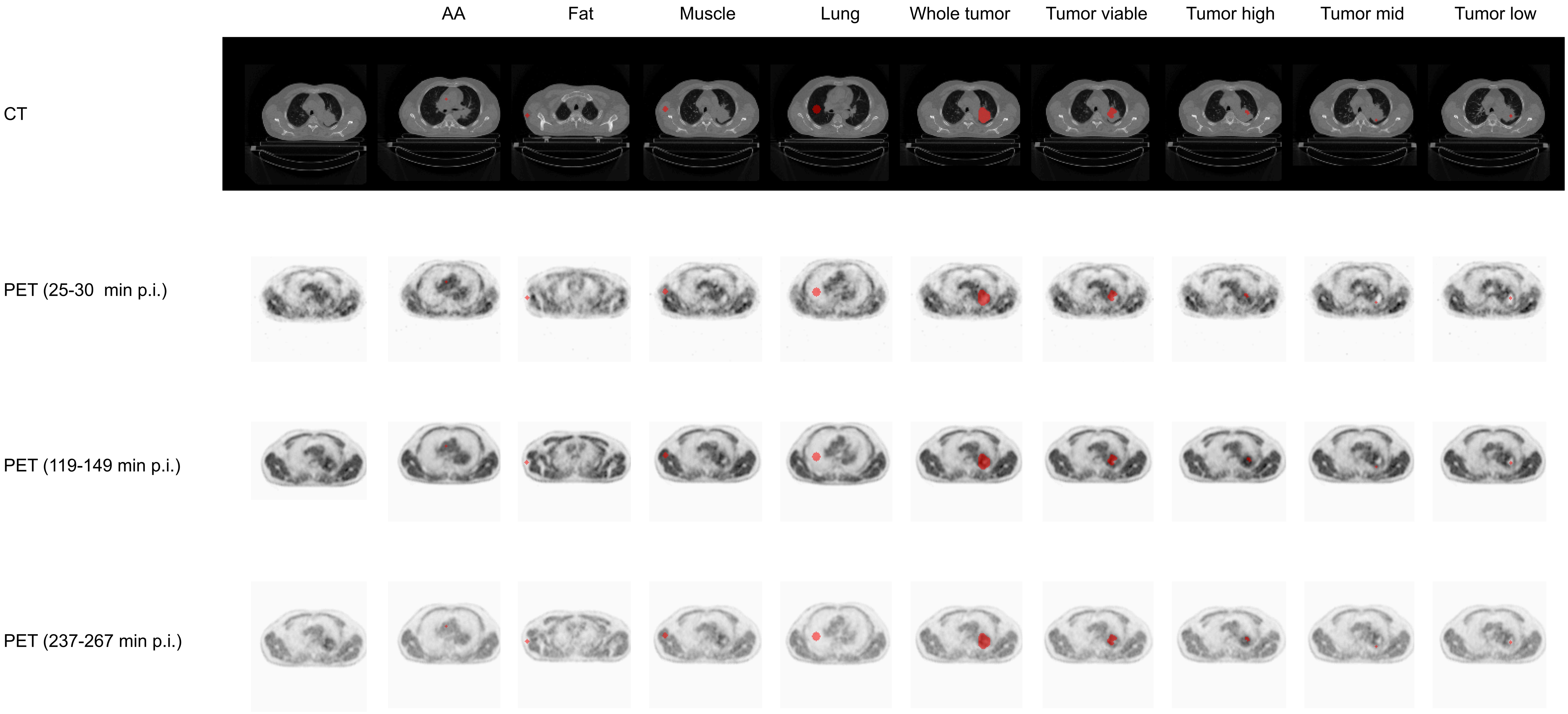

Supplement: Additional file 1: — Visualization of VOI for the typical images displayed in Fig. 1. (PDF 3910 kb) [file 40658_2016_167_MOESM1_ESM.pdf]
