# Supplementary material for: Pharmacokinetic modeling of a novel hypoxia PET tracer [18F]HX4 in patients with non-small cell lung cancer
Source: EJNMMI Phys. 2016 Dec 12;3:30. doi: 10.1186/s40658-016-0167-y (PMC5153396; doi:10.1186/s40658-016-0167-y)
Supplement: Additional file 2: — Model preference (%) for [18F]HX4 kinetics in various tissues according to AIC, based on 4.5 h dynamic PET data. (PDF 59 kb) [file 40658_2016_167_MOESM2_ESM.pdf]

Additional Table 1. Model preference (%) for [^{18}F]HX4 kinetics in various tissues according to AIC, based on 4.5 h dynamic PET data.

	Model					
Tissue type	1T2k	2T3k	2T4k	1T2k+V_B	2T3k+V_B	2T4k+V_B
Fat	-	-	25	12.5	37.5	25
Lung	-	-	-	25	50	25
Muscle	-	-	37.5	25	37.5	-
Liver	-	25	75	-	-	-
Tumor	-	-	-	-	-	100
Tumor, Viable	-	-	-	-	-	100
Tumor, High	-	-	12.5	-	-	87.5
Tumor, Mid	-	-	37.5	-	25	37.5
Tumor, Low	-	-	25	-	50	25

For clarity, values of 0% are not shown.
